# Loss of IL-10 secretion by regulatory B lymphocytes in multiple sclerosis patients

**DOI:** 10.1186/1479-5876-9-S2-P25

**Published:** 2011-11-23

**Authors:** Laure Michel, Nicolas Degauque, Alexandra Garcia, Marion Salou, Annie Elong Ngono, Jean-Paul Soulillou, David Laplaud, Sophie Brouard

**Affiliations:** 1 INSERM U643, Nantes University, Nantes, France; 2 Neurology Dept., Nantes Hospital, Nantes, France

## Background

Recently, an alternative role of B cells has emerged. Regulatory B cells have been shown to down-regulate immune responses in mice and humans. These cells control disease progression in several models of human autoimmune diseases, including a model of Multiple Sclerosis (MS). Their regulatory function mostly relies on their capacity to produce IL-10.

## Objectives

Our aim was to explore the frequency, the phenotype and the functional properties of regulatory B cells in MS patients compared to Healthy Volunteers (HV).

## Methods

All patients suffered from MS and had not received immune drugs since at least 6 months. The frequency and the phenotype of B cell subsets have been analysed using different specific markers: CD19^+^CD27^+^ memory B cells, CD19^+^CD38^dim^CD24^dim^ mature naïve B cells and CD19^+^CD24^high^CD38^high^ transitional B cells. Their capacity to secrete IL-10 was analysed *in vitro* 48 hours after stimulation by CD40 ligand and CpG ODN.

## Results

Thirty-eight MS patients (mean age: 41.55±12.8 yrs) and 21 HV (mean age: 35.4±13.4 yrs, NS) have been included. The patients had different MS forms: Clinically Isolated Syndrome (n=5), Relapsing-Remitting (n=23), Secondary Progressive (n=4) and Primary Progressive (n=6).

No significant difference was found for the frequency of the different B cell subsets in the different subgroups of patients. Particularly, MS patients harbor the same number (in frequency and absolute value) of CD19^+^CD24^high^CD38^high^ transitional B cells. However, the frequency of IL-10 secreting B cells was significantly decreased in MS patients (0.9±0.5%, n=9) compared to HV (2±0.95%, n=10, p<0.05, Mann Whitney test).

## Discussion/perspectives

MS patients display the same frequency of CD19^+^CD24^high^CD38^high^ transitional B cells, described as having regulatory properties. Nevertheless, B cells of MS patients present a significant decreased secretion of IL-10, supporting a defect in the B cells regulatory property. If confirmed, these results could have a considerable impact for the development of new therapeutic strategies.

